# Supplementary material for: SARS-CoV-2 mRNA Vaccines Elicit Different Responses in Immunologically Naïve and Pre-Immune Humans
Source: Front Immunol. 2021 Sep 27;12:728021. doi: 10.3389/fimmu.2021.728021 (PMC8502960; doi:10.3389/fimmu.2021.728021)
Supplement: Supplementary file 1 [file DataSheet_1.docx]

Supplementary Material

**Table S1. Demographic and serological information for the SPARTA participants in this study.** Dates for pre-, mid-, and post-vaccination timepoints are also noted. Antibody concentration refers to anti-RBD IgG antibodies, VN titer represents the reciprocal of the highest dilution point where cytopathic effects were not yet visible. (A) Participants from the immunologically naïve group who have no confirmed SARS-CoV-2 infection prior to vaccination. A single participant, P-032 had serum drawn between the two vaccinations. (B) Participants from the pre-immune group who have had confirmed SARS-CoV-2 infection prior to vaccination. 8 participants had serum drawn between the two vaccinations. (C) An additional 12 immunologically naïve participants who had serum collected between the two vaccinations.

**Table S2. Demographic and serological information for the validation cohort.** Antibody concentration refers to anti-RBD IgG antibodies, VN titer represents the reciprocal of the highest dilution point where cytopathic effects were not yet visible.


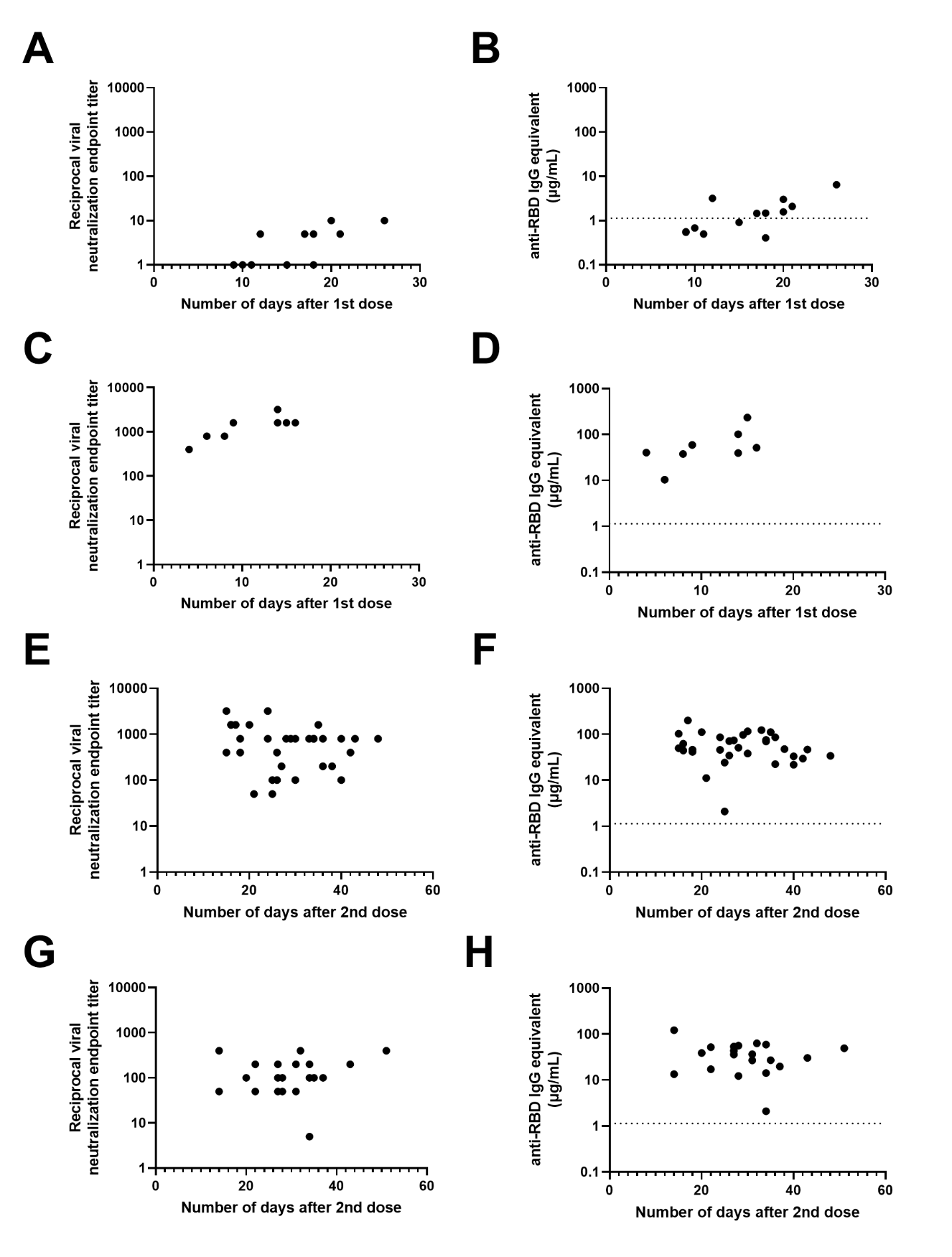


**Fig. S1: Antibody response based on number of days after the reception of the first and second vaccinations.** In immunologically naïve participants, (A) neutralizing and (B) anti-RBD IgG antibody levels showed a positive correlation with the number of days after the first vaccination the serum was collected (*r*=0.7781, ** *p*=0.0017, and *r*=0.6861, ** *p*=0.0096 respectively). In infected participants, (C) neutralizing antibody levels showed no correlation, with the number of days after the first vaccination the serum was collected (*r*=0.5319, *p*=0.1749), but (D) binding antibody levels did show a slight positive correlation (*r*=0.7085, * *p*=0.0492). In immunologically naïve participants, (E) neutralizing and (F) binding antibody levels showed no correlation with the number of days after the second vaccination the serum was collected (*r*=-0.3313, *p*=0.064, and *r*=-0.2190, *p*=0.2284 respectively). Similarly, in infected participants, neither (G) neutralizing nor (H) binding antibody levels showed any correlation with the number of days after the second vaccination the serum was collected (*r*=0.2068, *p*=0.3817, and *r*=-0.2458, *p*=0.2963 respectively). In order to represent the values on a logarithmic scale, lack of neutralization was reported as 1.
